# The Importance of MicroRNA Expression in Pseudoexfoliation Syndrome

**DOI:** 10.3390/ijms232113234

**Published:** 2022-10-31

**Authors:** Martyna Tomczyk-Socha, Wojciech Tomczak, Anna Turno-Kręcicka

**Affiliations:** 1 Department of Ophthalmology, Wroclaw Medical University, 50-556 Wroclaw, Poland; 2 Students’ Scientific Society, Department of Ophthalmology, Wroclaw Medical University, 50-556 Wroclaw, Poland

**Keywords:** microRNA, miR, pseudoexfoliation syndrome, etiopathogenesis

## Abstract

Pseudoexfoliation syndrome (PEX) is an important systemic disorder of the extracellular matrix, in which granular amyloid-like protein fibers accumulate in the anterior segment of the eyeball as well as in other organs. PEX is currently considered to be a multifactorial systemic disorder with genetic and environmental risk factors. The aim of this manuscript was to analyze miR expression in PEX. In recent years, an attempt has been made to investigate and describe the level of expression of selected miRs in PEX. Four polymorphisms of genes isolated from the blood that may be related to PEX were identified and miR-122-5p was found to be upregulated in patient blood. Furthermore, 18 miRs were identified with a statistically different expression in the aqueous humor. A significantly elevated expression of miR-125b was found in the anterior lens capsule, and four miRs were described, which may have a significant impact on the development of PEX. Regulatory miR molecules are gaining more and more importance in research aimed at identifying and isolating molecular markers related to the pathogenesis and prognosis of PEX, but further studies are needed.

## 1. Introduction

Pseudoexfoliation syndrome (PEX) is an important systemic disorder of the extracellular matrix. It is an age-related disease in which there is an accumulation of microscopic granular amyloid-like protein fibers in the anterior segment of the eyeball as well as in other organs including the heart muscle, lungs, connective tissue, blood vessels, skin, gallbladder, kidneys, liver, bladder, and meninges, making PEX a systemic disease [1,2,3].

PEX is currently considered to be a multifactorial systemic disorder with genetic and environmental risk factors. Molecular pathophysiology involves a complex interaction between pro-fibrotic protagonists (such as growth factors, proteolytic enzymes and inhibitors, pro-inflammatory cytokines, and chaperones) and dysregulated stress response pathways [4,5,6]. The interplay between genetically determined predisposing factors and stressors is a plausible theory explaining the development of PEX in the aging process.

The origin of the pseudoexfoliation material (PEXM) is associated with a dysregulation in elastin synthesis, the formation of abnormal elastotic fibers aggregates, and is accompanied by a significant reduction of collagen fibers [2]. While the structure of PEXM, the cells involved in its production, and histopathological changes at the tissue level have been characterized in detail, the exact pathological mechanisms underlying PEX formation remain unclear.

## 2. MicroRNA—Structure, Functions, and Importance

MicroRNAs (miRs) are single-stranded, non-coding, endogenous regulatory molecules. They are usually made of 21–23 nucleotides and arise from double-stranded precursors. They are present in human, animal, and plant cells. miRs represent a leading class of gene regulators [7,8,9]. The mechanism of miR molecules’ action is related to the posttranscriptional regulation of gene expression, which is possible due to the complementary binding with messenger RNA molecules [10]. miRs usually act as negative modulators of gene expression, but may also stimulate translation in some cases [11,12]. miR genes are most often arranged in clusters that are transcribed as polycistronic transcription units [13]. When they occur between protein-coding sequences, they function as independent transcriptional units. They can also be located in coding sequences. Genes for miRs are found in exons, introns, and untranslated regions [14]. Thanks to this arrangement of the transcription unit, the simultaneous formation of miR and mRNA transcripts is possible [15].

The formation of a miR consists of several stages that initially occur in the cell nucleus, and then in the cytoplasm. Transcription leads to the formation of the primary microRNA (pri-miR) transcript. The next step is pri-miR processing, which produces pre-miR. The resulting molecule is transferred to the cytoplasm. In the cytoplasm, the processing of pre-miR by the enzyme Dicer produces a double-stranded molecule approximately 20 nucleotides in length, which is then processed to produce a mature, functional miR. The active form of miR is ssRNA, embedded into the protein complex of miRNP, called RISC (microRNA-induced silencing complex) [13,15,16]. Mature miRs have a long half-life, are not rapidly degraded, and can affect various biological pathways by binding to different mRNAs [17].

## 3. The Role of miRs in the Pathogenesis and Development of Diseases

Numerous scientific reports prove that miRs play a very important role in course of many processes, such as cell division, cell differentiation, apoptosis, angiogenesis, oncogenesis, and the aging process [18].

Disturbances in miR expression are associated with various types of cancer. Almost 50% of the miR genes are located in fragile sites of the genome. Mutations in these areas are often associated with neoplasm, the formation of new cells, tissues, and substances. This indicates a significant role of miRs in the formation, development, and progression of many diseases, including cancer and other diseases in which new material is produced, such as in PEX [19].

The expression of microRNA was assessed in many neurodegenerative diseases, such as Alzheimer’s disease, amyotrophic lateral sclerosis, age-related macular degeneration, ataxia, dementia, myotonic dystrophy, epilepsy, glaucoma, Huntington’s disease, multiple sclerosis, Parkinson’s disease, and prion disorders. Many authors described a functional overlap in miR expression across diseases and reported selected miRs, which were dysregulated most often. Juźwik et al. distinguish miR-9-5p, miR-21-5p, the miR-29 family, miR-132-3p, miR-124-3p, miR-146a-5p, miR-155-5p, and miR-223-3p [20]. Nguyen et al. identified the overlapping miR-128, miR-140-5p, miR-206, miR-326, and miR-155, associated with multiple etiological cellular mechanisms [21].

More than one hundred miRs were described as important in primary open-angle glaucoma (POAG). miRs were linked with maintaining the balance of the aqueous humor, the change in the trabecular meshwork, and the apoptosis of retinal ganglion cells [22]. The neutralizing activity of several miRs and the effects of downregulation of pro-inflammatory and pro-fibrotic signaling pathways, including nuclear factor kappa-light-chain-enhancer of activated B cells (NF-kB), transforming growth factor-beta 2 (TGF-β2), Wnt/β-Catenin, and PI3K/AKT were described [23]. A few attempts were made to use miR as a therapeutic strategy for regulating intraocular pressure. RNA-based therapies for glaucoma can offer a potential role to deliver disease-modifying molecules. For example, miR-18a-5p and miR-21-5p were described as promising candidates for therapeutic targets in POAG patients [24,25].

## 4. The Expression of microRNAs in Pseudoexfoliation Syndrome

A systematic search of the published research for medical subject headings (MeSH) “pseudoexfoliation syndrome” or “pseudoexfoliation” and “microRNA” or “miRNA” was conducted using the electronic online database PubMed. Seven studies were found.

The first article examining miR expression in PEX was published in 2018 [26]. Chatzikyriakidou et al. examined the expression level of 92 gene polymorphisms in over 450 patients. The results were compared with a control group of healthy volunteers and with the study groups with pseudoexfoliation glaucoma (PEXG) or POAG. The size of these two study groups was over five times smaller than that of the study group with PEX. The genetic material was isolated from blood. Three polymorphisms related to PEX were identified. It was found that rs11382316 (in the “seed region”—in nucleotides 2–8 from the 5’ end of miR-3161, variant TA), rs2155248 (in mature miR-1304, variant T), and rs28635903 (in the flanking regions of miR-1268a, variant C) have a protective effect in terms of the risk of PEX [26].

In addition, two polymorphisms in the 3′-UTR region of genes involved in miR biogenesis were found to be related to PEX or PEXG. The rs1057035 polymorphism in the 3′-UTR of the DICER gene was associated with a reduced risk of PEX, while the rs55671916 polymorphism in the 3′-UTR of the XPO5 gene with an increased risk of PEXG [26].

Also in 2018, Drewry et al. presented a differential analysis of miR expression in the aqueous humor of patients with POAG and PEXG [27]. The control group consisted of patients with cataracts. Researchers identified 298 miRs, of which they singled out those with an expression that differed significantly between the groups. Expression of miR-125b-5p was decreased in POAG compared to the control group, and expression of miR-302d-3p and miR-451a was increased. In the PEXG group, five miRs with significantly different expressions were detected, compared to the control group. The number of readings for miR-122-5p, miR-3144-3p, miR-320e, and miR-630 was significantly higher in the study group, while miR-320a was significantly lower. What is interesting is that only one miR differing in expression between the two types of glaucoma was detected. The expression of miR-302d-3p was significantly lower in the PEXG group compared to POAG [27].

In 2020, overexpression of miR-125b was described in patients with PEX. The study compared the expression of miR-125b in three study groups—patients with POAG, PEXG, and PEX. The material was isolated from the anterior lens capsule and collected during cataract phacoemulsification. It was also found that the expression of miR-125b was statistically significantly increased in smoking patients [28].

Also in 2020, miR-122-5p was found to be significantly upregulated as PEX progressed into later stages and into PEXG. Of all miRs identified to be significantly differentially expressed in PEX, the authors distinguished miRs that seem to be controlling TGF-β1, protein binding, and extracellular matrix-related processes. miR-124-3p, miR-424-5p, and miR-122-5p were upregulated in PEXG, targeting those three specific pathways. The material was isolated from blood. For miR profiling, PCR arrays were used [29].

In 2022, a study was published which, using next-generation sequencing, selected four miRs with statistically different expressions. An anterior lens capsule was collected during cataract extraction [30]. The edgeR package was used in the statistical analysis and four miRs were distinguished—miR-671-3p, miR374a-5p, miR-1307-5p, and miR-708-5p. These four miRs may play a significant role in the etiopathogenesis of PEX [30].

Two essential RNase III enzymes were investigated in PEXG in a Saudi cohort in 2022 [30]. Polymorphism rs3742330 in the gene DICER1 and rs10719 in DROSHA were investigated in patients’ blood using real-time PCR. A significant genetic association was reported between variant rs3742330 in DICER1 and PEXG [31].

Another report [31] investigated miR profiles of Korean patients with PEXG and normal-tension glaucoma compared to healthy individuals using aqueous humor samples. Ten downregulated miRs (miR-3156-5p, miR-4458, miR-6717-5p, miR-6728-5p, miR-6834-5p, miR-6864-5p, miR-6879-5p, miR-877-3p, miR-548e-3p, and miR-6777-5p) and two upregulated miRNAs (miR-30d-5p and miR-320a) in PEXG patients compared to the control were found. RNA sequencing was conducted for 26 samples extracted from the aqueous humor.

## 5. Discussion

In recent years, an attempt has been made to investigate and describe the expression level of selected miRs in pseudoexfoliation syndrome. Four polymorphisms of genes isolated from the blood that may be related to PEX were identified and miR-122-5p was found to be upregulated in patient blood [26,29,31]. Furthermore, 18 miRs were identified with statistically different expressions in the aqueous humor [27,32]. Significantly, elevated expression of miR-125b was found in the anterior lens capsule [28], and four miRs [30] were described, which may have a significant impact on the development of PEX (Table 1).

## 6. Material Tested in miR Expression

Analyzing the existing literature, the following were used in research: a fragment of the anterior lens capsule, material from the aqueous humor, and blood, with divergent results [26,27,28,29,30,31,32].

The lens capsule epithelium is a good material for PEX pathophysiology studies. The piece of the anterior lens capsule that is examined is the tissue from the central pupil area where PEX-specific molecules are deposited and bound into insoluble structures. The lens capsule is an avascular structure washed by an aqueous fluid and is influenced by the ingredients it contains. Direct contact of the capsule with PEXM is also important, as is the possible sharing of PEXM production by the lens capsule epithelium.

Seland, examining the lens capsule epithelium in PEX using an electron microscope, described a characteristic fibrous substance organized into circular areas in contact with the epithelium [33]. The fibers varied in size and ranged from 10 to 150 μm. In the deep epithelial layer, discoid laminae were visualized, the position of which corresponded to the circular areas of the fibrous substance. Moreover, the connection of the discoid laminae of the deep layer with the circumferential granular band (in the form of radial sectors with the appearance of paving stones) visible in the partially digested lens capsule was reported. The ultrastructure of the fine-fibrous material of the deep layer was indistinguishable from that of the superficial material. Similar observations were made by Sveinsson in his study of the lens capsule in electron microscopy [34].

In another study, Seland found that the PEXM accumulated on the lens surface had numerous smooth protrusions. Consequently, he identified pits on the epithelium corresponding to the location and size of the aforementioned protrusions [35]. The author of the study suggested that the findings he described indicate that epithelial cells were the source of the material.

Further studies confirmed the relationship between the production of PEXM and epithelial cells in the anterior segment of the eye. In addition to the lens capsule epithelium, a correlation has also been demonstrated with the epithelium of the ciliary body, iris, and corneal endothelial cells [36,37]. However, despite the strong evidence from electron microscopy imaging, the exact origins and mechanisms of PEXM formation have not yet been elucidated.

The epithelium of the lens capsule is exposed throughout life to harmful factors that contribute to the pathogenesis of PEX and cataracts, such as ultraviolet (UV) radiation. Therefore, the lens capsule also appears to be an important model for studying complex factors that play a role in cell aging, including genetic and environmental influences due to its single-layer structure and direct exposure to UV radiation [38]. Both cataracts and PEX are age-related diseases, their risk factor is UV radiation, and their etiological mechanisms remain unclear.

Extracellular, circulating miRs are isolated from the aqueous humor and blood. miRs, unlike mRNAs, are resistant to endogenous and exogenous RNases, extreme temperatures, and pH levels. In blood, fresh-frozen tissues, paraffin blocks, and other secretions, miRs are very stable over a long period, even if left at room temperature. These features determine free molecules as perfect biomarkers [39,40].

Extracellular miRs are detectable in the blood serum in a replicable manner and their concentration is also similar in healthy people. Changes in the level of miR molecules may be a consequence of various physiological conditions, such as pregnancy (there are placental miRs in the blood serum of pregnant women that can be used to determine the age of pregnancy) viral infections, cancer, and many more diseases [41,42].

It should therefore be specified which material should be tested in PEX and compare miR expression by collecting aqueous humor, lens capsule, and blood from each patient to precisely assess the relationship between the profiles and the significant differences detected between particular miR expressions. Interestingly, Hindle et al. [43] demonstrated similar miR expression results for aqueous humor and blood. Unfortunately, a correlation between the expression of miR in the aqueous humor, lens capsule, and blood has not yet been investigated.

## 7. Study Limitations and Review Process Limitations

The described studies differed in the method used in the expression analysis, the size of the study group, the type of control group, and ethnicity. Each of these studies differed significantly from the others, which can also be seen in the results obtained. Due to the small number of studies performed on the pseudoexfoliation syndrome, it is not possible to determine which study is the most reliable. Therefore, further studies on a large number of participants are needed in order to be able to decide the risk of bias in the performed studies and their credibility in the future. In the last five years, we obtained the first results of miR expression in pseudoexfoliation syndrome, more are needed to clarify the role of miR in this disease.

## 8. Symptomatic PEX Diagnosis and the Need to Create a Diagnostic Test

PEX remains a disease for which diagnosis is based on symptoms. Only in an advanced stage, when PEXM deposits are visible on the lens capsule, can certainty be achieved and the diagnosis confirmed. Occasionally, iris translumination or high trabecular pigmentation can be seen during gonioscopy. In such conditions, PEX can only be suspected, as the described symptoms are not pathognomonic for PEX.

Additionally, there is a large group of patients who underwent lens removal before the onset of PEX symptoms. In these patients, we will never visualize the characteristic PEX lesions on the lens capsule. Therefore, the questions arise, how many people with a subluxated or dislocated lens without a history of trauma suffer from PEX, how many people diagnosed with primary open-angle glaucoma suffer from PEX-related secondary glaucoma, and how many people with intraoperative and postoperative complications suffer from PEX. Without a PEX-specific diagnostic test, these questions will remain unanswered.

Despite molecular research conducted so thoroughly and extensively over the past 20 years, there is still no answer to basic and the most intriguing questions about the etiology of PEX. Many researchers tried to understand the etiopathogenesis of PEX. They described many molecules, proteins, genes, and disrupted stress response pathways, all of which have a significant effect on PEX. However, it has not been possible to pinpoint the direct causal factor in PEX or link all the discoveries together into a single process.

## 9. Conclusions

Regulatory miR molecules are gaining more and more importance in research aimed at identifying and isolating molecular markers related to the pathogenesis and prognosis of various disorders. Knowledge of miR expression profiles in specific diseases may be used for a quick and precise diagnosis. Moreover, knowledge about disturbances in the expression of these molecules will probably determine the choice of therapy for individual patients.

## Figures and Tables

**Table 1:** Examined miR in pseudoexfoliation syndrome.

Authors	miR	Material	Examined Groups	Methods	Number of Patients
Chatzikyriakidou A. et al.	miR-3161, miR-1304, miR-1268a [26]	blood	PEX, PEXG, POAG	gene polymorphisms	569
Drewry M.D. et al.	miR-122-5p, miR-3144-3p, miR-320e, miR-630, miR-320a, miR-302d-3p [27]	aqueous humor	PEXG, POAG, patients with cataracts	NanoString technology, ddPCR	35,41
Tomczyk-Socha M. et al.	miR-125b [28]	anterior lens capsule	PEX, PEXG, POAG, patients with cataract	rt-PCR	150
Rao A. et al.	miR-124-3p, miR-424-5p, miR-122-5p [29]	blood	PEX, PEXG, ocular hypertension, control group	PCR array	50
Tomczyk-Socha M. et al.	miR-671-3p, miR374a-5p, miR-1307-5p miR-708-5p [30]	anterior lens capsule	PEX, patients with cataracts	NGS	10
Kondkar A.A. et al.	microRNA biogenesis gene—DICER1 [31]	blood	PEXG, healthy individuals	rt-PCR	680
Cho H.K. et al.	miR-3156-5p, miR-4458, miR-6717-5p, miR-6728-5p, miR-6834-5p, miR-6864-5p, miR-6879-5p, miR-877-3p, miR-548e-3p, miR-6777-5p, miR-30d-5p miR-320a [32]	aqueous humor	PEXG and normal-tension glaucoma, healthy individuals	RNA sequencing, rt-PCR	26

## Data Availability

The study did not report any data.
